# Awake uniportal VATS sublobar lung resections in high-comorbidity patients: Single-center early post-operative outcomes

**DOI:** 10.3389/fsurg.2023.1120414

**Published:** 2023-02-17

**Authors:** Giovanni Mugnaini, Domenico Viggiano, Paolo Fontanari, Rossella Forzini, Luca Voltolini, Alessandro Gonfiotti

**Affiliations:** ^1^ Thoracic Surgery Unit, Careggi University Hospital, Florence, Italy; ^2^ Department of Anhestesiology and Reanimation, Careggi University Hospital, Florence, Italy; ^3^ Department of Experimental and Clinical Medicine, University of Florence, Italy

**Keywords:** awake thoracic surgery, non-intubated thoracic surgery, spontaneous breathing, uniportal video assisted thoracic surgery, minimally invasive thoracic surgery, sublobar lung resections

## Abstract

**Introduction:**

Awake minimally invasive Uniportal Video Assisted Thoracic Surgery (U-VATS) represents the last challenge in thoracic surgery that could change the future scenario for high comorbidity patients with early-stage non-small cell lung cancer (NSCLC). We report a single center preliminary experience of awake thoracoscopic uni-portal anatomic and non-anatomic sub-lobar resections in this setting.

**Methods:**

We retrospectively analyzed data collected on a prospective database of patients undergoing U-VATS awake sub-lobar lung resections for NSCLC between September 2021 and September 2022. Inclusion criteria were clinical stage I disease; contraindication to standard lobectomy due to high respiratory function impairment; general anesthesia considered at high risk based on the American Society of Anesthesiologist score and on the Charlson Comorbidity Index. All patients underwent a standardized awake non-intubated anesthesia protocol approved by our institutional board.

**Results:**

They were *n* = 10 patients: *n* = 8 wedge resections; *n* = 2 segmentectomies. We had *n* = 1 (10%) conversion to standard general anesthesia and *n* = 1 laryngeal mask support but maintaining spontaneous breathing. *N* = 5 patients (50%) needed an Intensive Care Unit recovery (mean time = 17.20 h). Mean chest tube duration and Hospital stay were 2.0 and 3.5 days respectively. We did not register 30- days postoperative mortality.

**Conclusion:**

Awake thoracic surgery is a feasible technique, and it could be performed also in high comorbidities’ patients without a high rate of complications and allows to operate patients that so far were considered borderline for surgery.

## Introduction

Surgical interventions in spontaneous breathing patients, without mechanical ventilation and general anesthesia, have spread over the last two decades. Non-Intubated thoracic surgery (NITS) defined also as awake or tubeless thoracic surgery, and more specifically, non-intubated video-assisted thoracic surgery (NIVATS) have been increasingly adopted in thoracic surgery, also for complex lung resection, and it could potentially become the least minimally invasive procedure for lung resections.

General anesthesia with one-lung ventilation was mandatory for thoracic surgery until Pompeo et al. (1) had demonstrated the feasibility of lung resection in spontaneous breathing patients, first for wedge resection and lung volume reduction surgery (LVRS) (2–4) and then also for major resection (5). Nowadays, awake thoracic surgery has become a feasible surgical option that can also include patients with NSCLC or lung metastases who cannot undergo general anesthesia due to their high comorbidity.

The aim of this study is to show early postoperative outcomes of the awake lung resection in high comorbidity patients operated in our center in the last year and to demonstrate the feasibility of this technique also in this high-risk category.

## Materials and methods

We retrospectively analyzed data collected on a prospective database of patients undergoing U-VATS awake anatomic and non-anatomic sub-lobar lung resections for NSCLC in our center between September 2021 and September 2022. Inclusion criteria were: clinical stage I disease; contraindication to standard lobectomy due to high respiratory function impairment; general anesthesia considered at high risk based on the American Society of Anesthesiologist score (ASA score) and on the Charlson Comorbidity Index. The preoperative evaluation to consider a patient feasible for awake thoracic surgery is based on the latest literature exclusion criteria (6). All patients underwent conventional pre-operative examinations, including cardiological assessment and pulmonary function tests (PFTs), contrast enhanced thoracic and abdominal computed tomography scan (CT), brain CT scan and positron emission tomography-CT (PET-CT) scan. We performed an uniportal VATS resection, with a surgical access at the level of the anterior axillary line in the IV or in the V intercostal space, depending on the position of the lung lesion. The lesions were transected with endoscopic staplers. In the two segmental resections we performed an individual dissection of the pulmonary artery(ies), bronchus, and vein(s) and all these structures were transected with endoscopic staplers or by ligation and the use of energy devices. Every patient left the surgical theatre with a chest tube. The in-hospital post-operative evaluation consisted in monitoring a possible air leak and the 24 h amount of pleural liquid drained, as well as in executing a chest radiograph in post-operative day 1 and day 3 (unless patients were discharged before). All patients were referred to respiratory physiotherapy service from post-operative day 1 to the discharge. Preoperative characteristics were resumed in Table 1. We reported mean and median values of age, ASA score and CCI. Mean preoperative value of FEV1%, FVC% and DLCO% were 77.30%, 83.60% and 87.60% respectively.

**Table 1:** Patients’ preoperative characteristics. ASA: American Society of Anesthesiologists Classification; CCI: Charlson Comorbidity Index; COPD: Chronic Obstructive Pulmonary Disease; DM: Diabetes Mellitus; FEV1: Forced Expiratory Volume in 1 s; FVC: Forced Vital Capacity; DLCO: Diffusing Capacity for Carbon Monoxide; WR: Wedge Resection.

Patient's Preoperative Characteristics	
**Sex**	
Male	6
Female	4
**Age**	
Mean	68.60
Median	69.50
**Smoke history**	
Current	5
Former	3
Never	2
**ASA**	
WR 1	2
WR 2	3
WR 3	2
WR 4	2
WR 5	1
WR 6	1
WR 7	2
WR 8	2
S6 segm.	3
Lingulectomy	2
**ASA** (mean)	2.0
**CCI**	
WR 1	1
WR 2	3
WR 3	2
WR 4	2
WR 5	2
WR 6	1
WR 7	2
WR 8	2
S6 segm.	3
Lingulectomy	2
**CCI** (mean)	2.0
**Comorbidities**	
COPD	4
Cardiopathy	4
Arteriopathy	4
DM (I or II)	5
Others	6
**FEV1% pre**	
Mean	77.30
Median	80.50
**FVC% pre**	
Mean	83.60
Median	86.50
**DLCO% pre**	
Mean	87.60
Median	88.00

A written consent for the procedure was obtained from all patients.

### Anesthesiologic protocol for awake thoracic surgery

In collaboration with the anesthesiologist of our center (SOD Anestesia e Rianimazione, AOUC), for awake lung resection we developed a protocol that could lead to a safe surgical intervention and that could protect patients from severe hypoxia and hypercapnia, avoiding making them feel pain during surgical resections and reducing the cough reflex, allowing surgeons to operate and manipulate the lung in a safer way. We never performed the block of the vagus nerve. Airway nebulization of lidocaine and atomization of the lung and of the ilum failed in controlling the cough reflex in only one patient (10%). Table 2 shows protocol steps.

**Table 2:** Careggi university hospital anesthesiology protocol for awake lung resections. VM: VentiMask.

Anesthesiology protocol for Awake Thoracic Surgery
Premedication before starting sedation (15 min before):
• Atropine 0.01 mg/Kg e.v. • Dihydrocodeine 15 gtt per os
Sedation:
• VM with reservoir (6–10 L/min O2) to maintain SpO2 > 92% • Propofol 1–2 mg/Kg/h • Remifentanil 0.03–0.07 mcg/kg/min
Ultrasound guided paravertebral blockade: ESP (Erector Spinal Block) with ropivacaine 0.5%, 20–30 ml
In supine position, at least 30 min before surgery:
• Airway nebulization with lidocaine 2% (5 ml on high flow O2 with aerosol kit
Advanced patient monitoring with BIS™ (Bispectral Index)
Surgical incision infiltration with lidocaine 2%
Just before the surgical resection:
• Lidocaine (2%) atomization on the lung surface and on the hilum
Place a Laryngeal Mask if needed due to severe hypoxia and/or Hypercapnia

## Results

Our database reported that 10 patients underwent U-VATS awake resections between September 2021 and September 2022; *n* = 8 (80%) wedge resections (two of the RUL, three of the RLL, one of the LUL and two of the LLL) and *n* = 2 (20%) segmentectomies (one lingulectomy and one S6 left segmentectomy) were performed. Table 3 shows our results. We reported mean and median values. Among wedge resections, *n* = 1 patient (12.5%) required conversion to general anesthesia with orotracheal intubation due to difficulty controlled parenchymal bleeding and *n* = 4 patients (50%) required post-operative ICU monitoring due to their comorbidity. The mean time in ICU (hours) for the *n* = 4 four wedge resections who needed it was 17.50 h. There were no post-operative major complications, no prolonged air leak, and no infection and only the patient who needed conversion to general anesthesia needed a prolonged O2 support in the post-operative period. In the wedge resection group, the mean and the median time of maintenance of the chest tube were respectively 2.0 and 1.8 days and the mean and the median length of the hospitalization were 2.9 and 2.5 respectively.

**Table 3:** Early post-operative outcomes. ICU: Intensive Care Unit; WR: Wedge Resection.

	Length of stay	Chest tube duration (days)	Post-op ICU (hours)	Intra-op Complications	Post-op complications	30-days mortality
**WR 1**	2	1.8	20	None	None	–
**WR 2**	4	2	0	None	None	–
**WR 3**	2	1.6	0	none	None	–
**WR 4**	5	2.7	15	Conversion to general anesthesia due to bleeding	Prolonged O2 support needed	–
**WR 5**	3	0.8	0	None	None	–
**WR 6**	3	3	0	None	None	–
**WR 7**	2	1.8	17	None	None	–
**WR 8**	2	1.2	**18**	None	None	–
**Mean**	**2.9**	**2.0**	**8.8** **(17.50)**			
**Median**	**2.5**	**1.8**	**7.5** **(17.50)**			
**Left S6 segmentectomy**	8	2	16	None	Sputum retention	–
**lingulectomy**	4	2	0	Laryngeal mask needed (with spontaneous breath) due to uncontrolled cough reflex	none	–
**Mean**	3.5	**2.0**	**8.6** **(17.20)**			
**Median**	3	**2**	**7.5** **(17.00)**			

**Figure 1:**
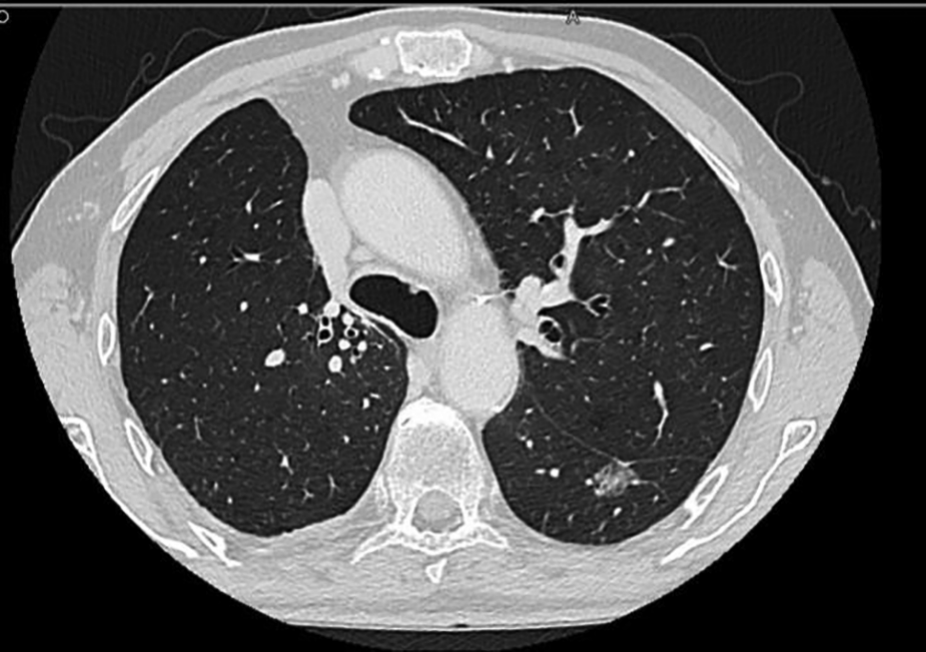
Pre-operative CT-scan of the left S6 segmentectomy showing the lesion.

**Figure 2:**
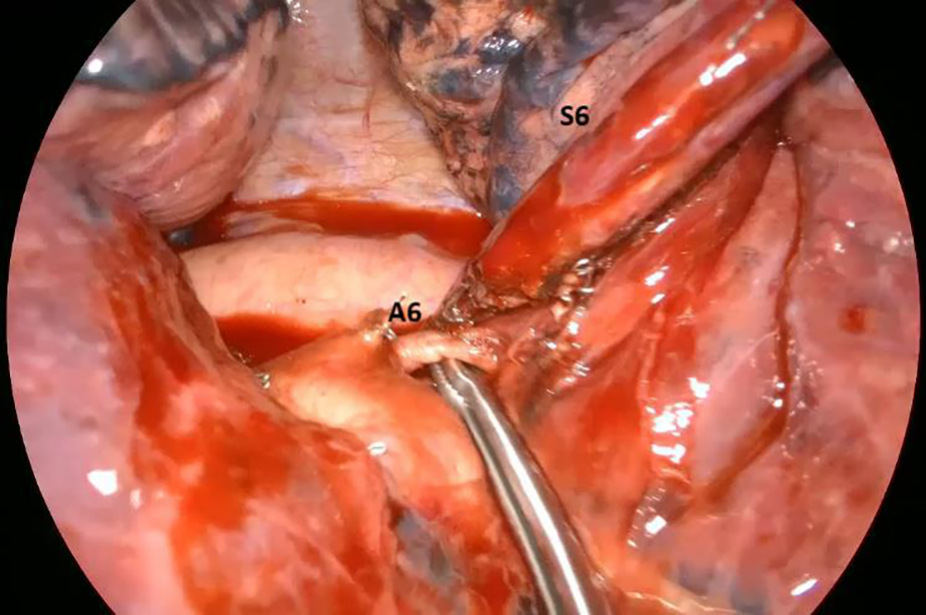
Intra-operative image showing the artery (A6) for the apical segment of the left lower lobe (S6).

The S6 left segmentectomy was a high comorbidity patient with a definitive tracheostomy due to a previous squamous carcinoma of the larynx, treated with neoadjuvant RT and CHT and then with a partial laryngectomy. He had a lesion of the left S6 suspected of NSCLC (and then confirmed by the pathological post-operative analysis, Figure 1), and we chose to perform a S6 segmentectomy to have good resection margin (Figure 2, Supplementary Video S1). This patient had no intraoperative complication but needed a post-operative monitoring in ICU (16 h) because of his comorbidities. He maintained the chest tube for 2 days. His hospitalization (8 days) was prolonged because of sputum retention due to the presence of the tracheostomy, and he underwent two post-op disobstructive bronchoscopies. The lingulectomy needed a laryngeal mask during the operation because of an uncontrolled cough reflex that did not allow a safe resection. The laryngeal mask enabled the anesthesiologist to control the cough reflex, the airway and the sO2 keeping the patient breathing spontaneously, without the use of muscle relaxants. The positioning of the parenchymal stapler required a controlled apnea (<1 min). He had no post operative complications and maintained the chest tube for two days. Among all patients who needed an ICU monitoring (*n* = 5, 50%), the mean time of ICU was of 17.20 h. The global mean time of ICU was 8.6 h. The global mean value of length of hospitalization and maintenance of the chest tube were respectively 3.5 and 2.0 days (Table 3). We did not register 30-days post-operative mortality.

## Discussion

### Spontaneous ventilation vs. general anesthesia

General anesthesia with one lung ventilation was considered necessary for lung resections, mainly because the surgeon can operate with non-ventilated lung and no breathing expansion. However, it has been demonstrated that general anesthesia and mechanical ventilation have a large series of potential side effects that could influence patients’ post operative morbidity and mortality, their length of the hospital stay and post-operative quality of life. Indeed, mechanical ventilation could lead to a lung damage because of an airway pressure-induced injury, atelectasis in the non-ventilated lung, the release of pro-inflammatory mediators (7–10). General anesthesia, use of muscle relaxant and use of opioids could cause a prolonged hospital stay and an augmented risk of mortality and morbidity and post operative cognitive dysfunction (11–13). In addition, orotracheal and bronchial intubation have potential, not insignificant, complications such as post-op throat pain or laryngeal and/or tracheal injuries (14).

Surgical pneumothorax in non-intubated thoracic surgery requires the anesthesiologist to manage the paradoxical ventilation and the risk of hypoxemia, due to a potential increase of the right-left intrapulmonary shunt. Hypoxic pulmonary vasoconstriction, a defense mechanism which is activated by the hypoxic alveoli, is more efficient during NITS, since we do not use some anesthetic drugs, like volatile anesthetics that can inhibit the protective vasomotor response of HPV (15). Furthermore, the anesthesiologist must avoid severe hypercapnia and acidosis during the awake thoracic surgery. Patients with a background of severe COPD or neuromuscular diseases have a higher risk for developing intraoperative hypercapnia. However, transient perioperative permissive hypercapnia has been well described (16).

Cough reflex, triggered by lung manipulation, could be the surgeon’s ‘enemy’ during NITS: in the last years several techniques were developed to reduce this reflex: inhalation of aerosolized lidocaine, application of a spray of lidocaine on the lung surface, a stellate ganglion block or a vagus nerve block placed intrathoracically were all feasible techniques (16).

Thoracic surgery in non-intubated patients was already known in the 1920s, when Jacobeaus began to use thoracoscopy in patients with a suspected tuberculosis or other intrathoracic diseases to make diagnosis and to perform cauterization of adhesions in awake patients (17). After all, mechanical ventilation was not introduced before the 1960s and patients’ survival after a thoracotomy in spontaneous ventilation was extremely low. At the end of the ‘50s, high number cases studies have been published reporting awake anesthesia techniques for major lung resections that have subsequently inspired current techniques (18, 19). With the introduction of the mechanical ventilation in early 1960s and mainly with the introduction of the double lumen tube, the one lung ventilation with a controlled general anesthesia became the gold standard for thoracic surgery. At the beginning of the XXI century, thoracic surgeons started to question themselves about the feasibility of lung resections avoiding general anesthesia. The first study was undertaken by Pompeo et al., who published a small randomization of 60 patients who underwent a wedge resection in general anesthesia (control group) or without it, with sole thoracic epidural anesthesia (awake group) and providing oxygen *via* a face mask. They demonstrated that the awake group had a significant reduced hospital stay, a greater anesthesia satisfaction score and a significantly lower post-operative *Δ*PaO2 (1). Since then, there has been a growing interest in this technique: only three years later, in 2007, Al-Abdullatief et al. published an observational study showing the possibility to perform awake anesthesia also in some cases of major thoracic resection, emphasizing above all the importance of avoiding muscle relaxants during thymectomies in patients with myasthenia gravis (20). In 2012, Chen et Al. published an important work showing a 3-years’ experience of non-intubated lung resection with 285 cases, of which 159 (55.8%) patients for primary lung cancer. They reported only fourteen (4.9%) conversion to tracheal intubation and no perioperative mortality (21). First applied for wedge resection, lung biopsies, metastasectomies (22) and lung volume reduction surgery (23, 24), the awake technique had a high development in the last years and recent studies reported the use of awake anesthesia for major lung resection (5) and even during thoracotomy (25), without significant differences in terms of survival and post-operative mortality, but with a faster recovery after surgery and a low rate of conversion to general anesthesia (0%–3%) (26). Awake lung biopsies still play a leading role in diagnosis of interstitial lung disease although the increasing diffusion of cryobiopsy, that still have a lower diagnostic yield compared to surgery (27).

In this study we reported a single center’ preliminary experience and early post-operative outcomes of awake U-VATS sublobar lung resection performed between September 2021 and September 2022. We performed *n* = 8 wedge resections and *n* = 2 segmentectomies (one lingulectomy and one S6 left segmentectomy) for NSCLC, without post-operative major complications. Only *n* = 5 (50%) patients needed an ICU post-operative monitoring with a mean time of 17.20 h. We reported length of stay and chest tube duration values higher than other results in literature (26), mainly because we selected high comorbidities’ patients for our awake lung resections. Indeed, this kind of patients would have been difficult to enroll for lung surgery under general anesthesia, due to the higher risk of post-operative complications, morbidity, and mortality.

This study has several limits: first, it is a single-center retrospective analysis of a small cohort of high-selected patients. Reporting early post operative outcomes, we cannot include overall and event-free survival in our results. We are carrying out a larger follow-up of these patients and updated results may be published in the future.

## Conclusions

This work confirms what has been reported in literature and given our results (although relating to a limited number of cases) we can assume that awake U-VATS sub-lobar surgery is a feasible technique and a viable option to the well-known VATS under general anesthesia and could represent an innovative strategy in high comorbidities NSCLC patients traditionally considered at high risk for anatomical resections under general anesthesia.

## Data Availability

The raw data supporting the conclusions of this article will be made available by the authors, without undue reservation.

## References

[1] Pompeo E Mineo D Rogliani P Sabato AF Mineo TC. Feasibility and results of awake thoracoscopic resection of solitary pulmonary nodules. Ann Thorac Surg. (2004) 78(5):1761–8. 10.1016/j.athoracsur.2004.05.083 15511470

[2] Pompeo E Rogliani P Palombi L Orlandi A Cristino B Dauri M. Awake thoracic surgery research group (ATSRG). the complex care of severe emphysema: role of awake lung volume reduction surgery. Ann Transl Med. (2015) 3(8):108. 10.3978/j.issn.2305-5839.2015.04.17 26046049 PMC4436426

[3] Pompeo E Rogliani P Tacconi F Dauri M Saltini C Novelli G Awake thoracic surgery research group. Randomized comparison of awake nonresectional versus nonawake resectional lung volume reduction surgery. J Thorac Cardiovasc Surg. (2012 Jan) 143(1):47–54.54.e1. 10.1016/j.jtcvs.2011.09.050 22056369

[4] Pompeo E Tacconi F Mineo TC. Comparative results of non-resectional lung volume reduction performed by awake or non-awake anesthesia. Eur J Cardiothorac Surg. (2011) 39(4):e51–8. 10.1016/j.ejcts.2010.11.071 21397783

[5] Gonzalez-Rivas D Fernandez R de la Torre M Rodriguez JL Fontan L Molina F. Single-port thoracoscopic lobectomy in a nonintubated patient: the least invasive procedure for major lung resection? Interact Cardiovasc Thorac Surg. (2014) 19(4):552–5. 10.1093/icvts/ivu209 25006214

[6] Gonzalez-Rivas D Bonome C Fieira E Aymerich H Fernandez R Delgado M Non-intubated video-assisted thoracoscopic lung resections: the future of thoracic surgery? Eur J Cardiothorac Surg. (2016) 49(3):721–31. 10.1093/ejcts/ezv136 25896196

[7] Della Rocca G Coccia C. Acute lung injury in thoracic surgery. Curr Opin Anaesthesiol. (2013) 26:40–6. 10.1097/ACO.0b013e32835c4ea2 23235524

[8] Schilling T Kozian A Huth C Buhling F Kretzschmar M Welte T The pulmonary immune effects of mechanical ventilation in patients undergoing thoracic surgery. Anesth Analg. (2005) 101:957–6. 10.1213/01.ane.0000172112.02902.77 16192502

[9] Gothard J. Lung injury after thoracic surgery and one-lung ventilation. Curr Opin Anaesthesiol. (2006) 19:5–10. 10.1097/01.aco.0000192783.40021.c1 16547427

[10] Munoz JF Ibanez V Real MI Gonzalez O Munoz P German MJ High-frequency jet ventilation in thoracic surgery. Rev Esp Anestesiol Reanim. (1998) 45:353–4. PMID: . 9847648

[11] Sessler DI Sigl JC Kelley SD Chamoun NG Manberg PJ Saager L Hospital stay and mortality are increased in patients having a “triple low” of low blood pressure, low bispectral index, and low minimum alveolar concentration of volatile anesthesia. Anesthesiology. (2012) 116:1195–203. 10.1097/ALN.0b013e31825683dc 22546967

[12] Murphy GS Szokol JW Avram MJ Greenberg SB Shear T Vender JS Postoperative residual neuromuscular blockade is associated with impaired clinical recovery. Anesth Analg. (2013) 117:133–41. 10.1213/ANE.0b013e3182742e75 23337416

[13] Hausman MS Jr Jewell ES Engoren M. Regional versus general anesthesia in surgical patients with chronic obstructive pulmonary disease: does avoiding general anesthesia reduce the risk of postoperative complications? Anesth Analg. (2015) 120:1405–12. 10.1213/ANE.0000000000000574 25526396

[14] Fitzmaurice BG Brodsky JB. Airway rupture from double-lumen tubes. J Cardiothorac Vasc Anesth. (1999) 13:322–9. 10.1016/S1053-0770(99)90273-2 10392687

[15] David P Pompeo E Fabbi E Dauri M. Surgical pneumothorax under spontaneous ventilation-effect on oxygenation and ventilation. Ann Transl Med. (2015) 3(8):106. 10.3978/j.issn.2305-5839.2015.03.53 26046047 PMC4436421

[16] Kiss G Castillo M. Nonintubated anesthesia in thoracic surgery: general issues. Ann Transl Med. (2015) 3(8):110. 10.3978/j.issn.2305-5839.2015.04.21 26046051 PMC4436416

[17] Jacobaeus HC. The cauterization of adhesions in artificial pneumothorax treatment of pulmonary tuberculosis under thoracoscopic control. Proc R Soc Med. (1923) 16:45–62. PMID: ; PMCID: . 19982912 10.1177/003591572301600506 PMC2103718

[18] Vischnevski AA. Local anesthesia in thoracic surgery: lungs, heart and esophagus. Minerva Anestesiol. (1954) 20:432–5. PMID: . 14355684

[19] Ossipov BK. Local anesthesia in thoracic surgery: 20 years experience with 3265 cases. Anesth Analg. (1960) 39:327–32. 10.1213/00000539-196007000-00012 13731463

[20] Al-Abdullatief M Wahood A Al-Shirawi N Arabi Y Wahba M Al-Jumah M Awake anaesthesia for major thoracic surgical procedures: an observational study. Eur J Cardiothorac Surg. (2007) 32(2):346–50. 10.1016/j.ejcts.2007.04.029 17580117

[21] Chen KC Cheng YJ Hung MH Tseng YD Chen JS. Nonintubated thoracoscopic lung resection: a 3-year experience with 285 cases in a single institution. J Thorac Dis. (2012) 4(4):347–51. 10.3978/j.issn.2072-1439.2012.08.07 22934136 PMC3426741

[22] Pompeo E Mineo TC. Awake pulmonary metastasectomy. J Thorac Cardiovasc Surg. (2007) 133(4):960–6. 10.1016/j.jtcvs.2006.09.078 17382634

[23] Mineo TC Pompeo E Mineo D Tacconi F Marino M Sabato AF. Awake nonresectional lung volume reduction surgery. Ann Surg. (2006) 243(1):131–6. 10.1097/01.sla.0000182917.39534.2c 16371748 PMC1449981

[24] Pompeo E Elkhouly A Rogliani P Dauri M Peer M Sergiacomi G Quasilobar minimalist lung volume reduction surgery. Eur J Cardiothorac Surg. (2021) 60(3):598–606. 10.1093/ejcts/ezab174 33860323

[25] Furák J Szabó Z Tánczos T Paszt A Rieth A Németh T Conversion method to manage surgical difficulties in non-intubated uniportal video-assisted thoracic surgery for major lung resection: simple thoracotomy without intubation. J Thorac Dis. (2020) 12(5):2061–9. 10.21037/jtd-19-3830 32642108 PMC7330381

[26] Szabo Z Fabo C Oszlanyi A Hawchar F Géczi T Lantos J Anesthetic (r)evolution from the conventional concept to the minimally invasive techniques in thoracic surgery-narrative review. J Thorac Dis. (2022) 14(8):3045–60. 10.21037/jtd-22-80 36071785 PMC9442516

[27] Korevaar DA Colella S Fally M Camuset J Colby TV Hagmeyer L European Respiratory Society guidelines on transbronchial lung cryobiopsy in the diagnosis of interstitial lung diseases [published online ahead of print, 2022 jun 16]. Eur Respir J. (2022) 60:2200425. 10.1183/13993003.00425-2022 35710261

